# Causal Biological Network Model for Inflammasome Signaling Applied for Interpreting Transcriptomic Changes in Various Inflammatory States

**DOI:** 10.1155/2022/4071472

**Published:** 2022-01-27

**Authors:** Hasmik Yepiskoposyan, Manuel C. Peitsch, Marja Talikka

**Affiliations:** PMI R&D, Philip Morris Products S.A., Quai Jeanrenaud 5, 2000 Neuchâtel, Switzerland

## Abstract

Virtually any stressor that alters the cellular homeostatic state may result in an inflammatory response. As a critical component of innate immunity, inflammasomes play a prominent role in the inflammatory response. The information on inflammasome biology is rapidly growing, thus creating the need for structuring it into a model that can help visualize and enhance the understanding of underlying biological processes. Causal biological network (CBN) models provide predictive power for novel disease mechanisms and treatment outcomes. We assembled the available literature information on inflammasome activation into the CBN model and scored it with publicly available transcriptomic datasets that address viral infection of the lungs, osteo- and rheumatoid arthritis, psoriasis, and aging. The scoring inferred pathway activation leading to NLRP3 inflammasome activation in these diverse conditions, demonstrating that the CBN model provides a platform for interpreting transcriptomic data in the context of inflammasome activation.

## 1. Introduction

The optimal functioning of a cell, a tissue, an organ, and, ultimately, an organism as a whole depends on homeostasis and damage mitigation. Any deviation of the homeostatic range leads to a stress response, and when the stress response is insufficient to restore homeostasis, an inflammatory response is engaged [1]. In the recent decade, it has become increasingly clear that inflammasomes are critical components of inflammatory response. Inflammasomes are multimolecular complexes that can assemble upon exposure to pathogen-associated molecular patterns (PAMP), damage-associated molecular patterns (DAMP), and diverse environmental insults [2]. Depending on the stress sensor molecule, inflammasomes can be of different types, such as NLRP1 (NLR family pyrin domain containing 1), NLRP2, NLRP3, NLRC4 (NLR family CARD domain containing 4 or IPAF), AIM2 (absent in melanoma 2), IFI16 (interferon-gamma inducible protein 16), and pyrin inflammasomes [3]. Inflammasome activation has been linked to viral, bacterial, and fungal infections [4–6] and autoinflammatory and autoimmune diseases such as gout and rheumatoid arthritis [7–9], as well as to other diverse conditions such as Alzheimer's disease, atherosclerosis, aging, or obesity [10–14].

Generally, the two-signal mediated inflammasome activation model is accepted and discussed in the literature. During the first signal—the priming signal—inflammasome component and interleukin gene expressions are increased downstream of the NF*κ*B (nuclear factor kappa-light-chain-enhancer of activated B cells) complex [15]. Consequently, the inflammasome is activated following diverse signals, which often culminate at second messengers such as reactive oxygen species (ROS), ATP, or potassium levels [16–18]. The canonical inflammasome complex assembly results in procaspase 1 cleavage into active caspase 1 (CASP1), which subsequently cleaves and thus activates the proinflammatory cytokines interleukin 1B (IL1B) and IL18 [19]. Cytokines play a crucial role in the innate immune response against invading pathogens; however, the inflammatory response can flare out of control. For example, exacerbated immune response as a result of NLRP3 inflammasome activation in pulmonary tissue can lead to acute lung injury and acute respiratory distress syndrome [20–22], which are common clinical manifestations of coronavirus disease 2019 (COVID-19). Indeed, studies show the engagement of the inflammasome in COVID-19 leading to uncontrolled inflammatory response and pyroptosis, an inflammatory form of cell death [23–25].

The complex biological state induced during an inflammatory response cannot be explained by analyzing isolated molecules or endpoints. While the information on the changes in separate molecular species is valuable, a holistic view that integrates committed inflammatory pathways and the interconnections between pathway molecules can provide enhanced understanding of the impacted biology. Compilation of the involved pathways in a network representation is a step toward comprehensive understanding of a system response. Such a network approach is a valuable tool in network medicine, which takes into account how the regulated molecules interact with each other and how information flows along the multiple pathways that constitute a given biological process or pathology [26, 27]. Organizing the established biological data into a network view can help link apparent unrelated processes and pathways, facilitating characterization of known or novel disease mechanisms and prediction of drug effects and treatment outcomes.

Furthermore, vast amounts of data generated through omics technologies, for example, transcriptomics, can be harnessed in concert with network models to maximize information acquisition. From the RNA expression values in transcriptomic datasets, the activity levels of their regulating factors can be extrapolated and used to score the entities of network models [28]. Such an approach creates a powerful explorative tool by combining literature-derived network models with billions of data points that are publicly available. This methodology conforms to the 21st century paradigm for medical research, which shifts the research from animal tests to assessments in cells and tissues in the context of systems biology with the use of in silico tools [29, 30].

To characterize the mechanisms responsible for disease or toxicant exposure, we have published a series of causal biological network (CBN) models that capture unstructured information from scientific literature into scorable graphical representations. The network models were built using Biological Expression Language (BEL) [31], which converts molecular interactions described in natural language to semantic triples with source, relationship, and target by using a controlled vocabulary. This allows us to compute the triples into a graphical representation in which the nodes represent biological entities and the edges represent the interactions between the nodes. BEL also captures information on the experimental approach, including species, tissue/cell type, and disease state. The models are hosted in the CBN database (http://causalbionet.com) [32, 33]. The newest additions in CBN are a suite of models that describe mucociliary clearance [34], a zebrafish cardiotoxicity network model for ecotoxicology [35], and a suite of models that represent signaling pathways that contribute to inflammatory bowel disease [36]. The website offers tools for browsing and downloading each network model. Finally, the network models can be scored using transcriptomic data to obtain genome-wide coverage of mRNA changes in the context of a biological process. The scoring principle is described in Materials and Methods and in several publications [37–41].

## 2. Materials and Methods

### 2.1. Literature Curation

Relevant scientific literature containing mechanistic data on inflammasome activation was identified as the first step in network building. Research articles were prioritized over review articles, and results sections were prioritized over the introduction, discussion, and conclusion sections in order to capture direct experimental evidence. Wherever applicable, the most granular sequence of causal events was captured. Scientific text curation was performed with BEL, which displays biological findings in a computable form. BEL converts the relationships between biological entities into statements of cause, relationship, and effect triples using controlled vocabularies facilitating the subsequent computation of the models. Biological entities of the cause and target are scripted in a namespace format of “BEL function (namespace identifier:“entity definition”),” for example, “p(HGNC:NLRP3).” Custom namespaces (e.g., PMIBP for a biological process) were created if the biological entities were not available in existing namespaces.  Table 1 summarizes the BEL terminologies for the namespaces used in this study.

The namespaces were connected with causal or associative relationships based on literature evidence. The publication reference and details of the experimental model (such as species, organ/tissue/cell types, or cell lines) were annotated additionally to provide context to the statements. Contradicting evidence was curated without preferential treatment, along with experimental model annotations. The BEL statements for the inflammasome network model were derived from experiments conducted in human, mouse, and rat model systems. To avoid duplicate namespaces for the same gene curated from studies in different species, we orthologized the network model to single species nomenclature.

### 2.2. Network Model Assembly and Scoring

In order to generate a network view, BEL statements were compiled into a network assembly model by using OpenBEL framework 3.0.0 (https://github.com/OpenBEL/openbel-framework). The Cytoscape web application was used to visualize and analyze network properties [42]. The model backbone consists of nodes, which are the biological entities connected by relationship edges. In addition to the backbone layer, the network model has a downstream layer that harbors information about mRNAs regulated by some of the entities in the model backbone. The downstream layer is based on a back-reasoning approach, where the activity of a backbone node is deduced from its own downstream transcript abundance [28]. This collective mRNA signature was obtained from publicly available transcriptomic datasets. The activity of the nodes in the model backbone is inferred on the basis of the concordance of transcriptomic changes in the data (chosen datasets for scoring and analysis) with the mRNA nodes underneath the backbone nodes. Such an approach allows one to infer the activity status of the corresponding backbone node (inferred node: iNode) from scoring datasets instead of assuming that the mRNA abundance of the backbone node in the dataset corresponds with its protein activity. The datasets GSE51386, GSE55235, GSE2737, E-MEXP-839, and GSE11258 from public data repositories were used for scoring the inflammasome network model. For model scoring, a threshold-free enrichment method specific for iNode was applied using the Strength Network Perturbation Amplitude scoring algorithm [43]. To illustrate graphically how backbone nodes are inferred on the basis of gene expression differences in the scoring dataset, the inference values were imported to the Cytoscape application and assigned to the model nodes.

## 3. Results and Discussion

### 3.1. The Inflammasome Model

In this work, we have built a CBN model for inflammasome activation. We curated scientific literature related to inflammasome activation from 55 research articles and compiled a network model with 297 nodes and 455 edges, which can be used to score the pathology of infections, diseases, treatments, and other conditions. The network model is centered on NLRP3 inflammasome activation, as it is the most studied and best characterized inflammasome. As inputs, the model contains nodes that represent toxins and various pathogenic and sterile insults. Toll-like receptors (TLR), sirtuin 1 (SIRT1), Beclin 1 (BECN1), NF*κ*B complex, aryl hydrocarbon receptor (AHR), Z-DNA binding protein 1 (ZBP1), and mitogen-activated protein kinase (MAPK) signaling are some of the upstream regulators of the NLRP3 inflammasome complex. Caspase 1 (CASP1) and the subsequent cleavage and activation of IL1B and IL18 are among the downstream targets of the NLRP3 inflammasome complex. The inflammasome network model is available for browsing and download in the CBN database (http://causalbionet.com/).

### 3.2. Network Model Scoring with Transcriptomic Data

#### 3.2.1. Mouse Lung Response to Viral Infection

To demonstrate how the inflammasome CBN model can be used to gain mechanistic insights into inflammatory response in tissues, we scored the network model with gene expression data from several publicly available datasets hosted in the Gene Expression Omnibus (GEO) repository. The transcriptomic dataset GSE51386 contains data on mouse lung response to severe acute respiratory syndrome (SARS) MA15 virus infection at 10^4^ PFU. The lungs from SARS MA15-and mock-infected C57BL6 mice were dissected at 4 and 7 days post infection (dpi). The mRNA abundances in affected and control lung cells were scored against our iNode collection, and the results of the scoring show whether a given iNode is inferred to be activated, inhibited, or not impacted.

**Figure 1:**
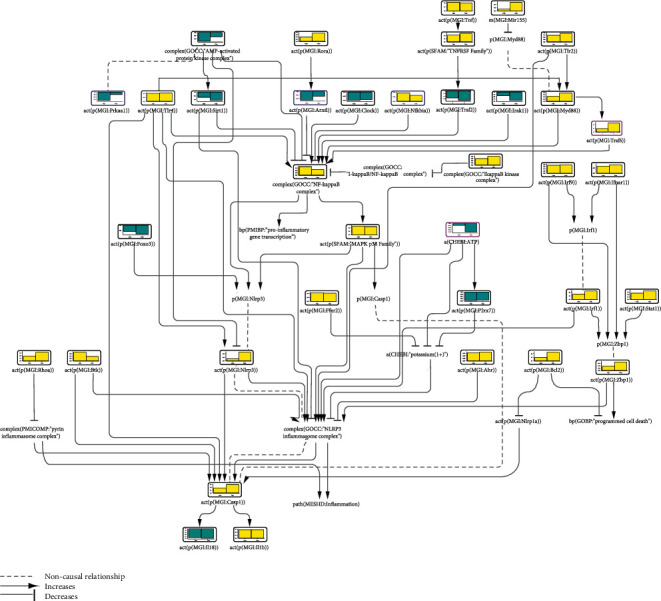
Part of the inflammasome network model scored with transcriptomic data from GSE51386. The bar graph above each node, which was scored, shows the inferred fold change in mouse lung samples at (1) 4 and (2) 7 days post infection (dpi) relative to uninfected lung samples at the same time point. The directionalities are shown as yellow or blue bars for inferred upregulation or downregulation, respectively. The black outline refers to nodes that were significantly impacted in both samples at both 4 and 7 dpi, the blue outline refers to nodes that were significantly impacted only at 4 dpi, and the purple outline refers to nodes that were significantly impacted only at 7 dpi.

The iNode scoring algorithm inferred upregulation of NLRP3 and CASP1 at both 4 and 7 dpi, with a greater effect at a later time point (Figure 1). These nodes, which are an integral part of the NLRP3 inflammasome complex, receive the activating input from multiple upstream factors. Particularly, members of the NF*κ*B pathway, including TLR2, TLR4, MYD88 (myeloid differentiation primary response 88), and the NF*κ*B complex, were inferred to be upregulated upon virus infection, consistent with the NLRP3 inflammasome priming event. TNF (tumor necrosis factor) and its receptor were also inferred to be upregulated, although TRAF2 (TNF receptor-associated factor 2), through which TNF connects to NF*κ*B in our CBN, was inferred to be significantly downregulated at both 4 and 7 dpi, which hinted at other parallel pathways involved in the infection-elicited inflammation. Furthermore, the AMPK (AMP-activated protein kinase) complex, a known inhibitory factor of NF*κ*B, was inferred to be downregulated. Similarly, SIRT1, which blocks NF*κ*B-DNA binding through deacetylation [44] and also functions as an inflammasome inhibitor, was also inferred to be downregulated. Some other inhibitory factors in the NF*κ*B cascade, such as NFKBIA and MIR155 upstream of MYD88, were inferred to be upregulated; however, it appeared that positive signals toward NF*κ*B overcame these inhibitory effects. Two circadian proteins, CLOCK and ARNTL (aryl hydrocarbon receptor nuclear translocator like, also known as BMAL1)—which have opposing effects on NF*κ*B—were both inferred to be downregulated. NF*κ*B upstream activator IRAK1 (interleukin 1 receptor-associated kinase 1) was also inferred to be downregulated. This complex regulation of NF*κ*B could be a fine-tuning mechanism of inflammation control. In this context, it is important to note that NF*κ*B was shown to also have inhibitory effects on NLRP3 inflammasome activation by promoting clearance of damaged mitochondria [45]. DAMPs released during mitochondrial damage, such as mitochondrial ROS and mitochondrial DNA, are recognized as triggers for NLRP3 activation [46].

Scoring with a SARS MA15 virus-treated dataset further showed that NLRP3 inflammasome-activating factors, such as FFAR2 (free fatty acid receptor 2), BTK (Bruton's tyrosine kinase), IRF1 (interferon regulatory factor 1), and ZBP1, were all inferred to be upregulated, although the inflammasome inhibitory AHR was also inferred to be upregulated. Inconsistent with its inflammasome-activating role, the purinergic receptor P2RX7 was inferred to be significantly downregulated at both time points after viral infection. The downstream effector molecules of inflammasome activation, IL1B and IL18, were inferred as significantly affected after virus infection. While, as expected, IL1B was inferred to be upregulated at both time points after viral infection, IL18 activity was inferred to be downregulated. This can be explained by signals other than NLRP3 inflammasome affecting IL18 activity. BCL2 apoptosis regulator and RHOA (ras homolog family member A)—inhibitors of NLRP1 activity and the pyrin inflammasome, respectively—were inferred to be upregulated, suggesting inhibition of both NLRP1 and pyrin inflammasome activities. In addition, various other factors are involved in IL18 production, such as JAK/STAT (Janus kinase/signal transducer and activator of transcription) or type I interferon signaling [47], and the alterations in these pathways can be reflected in the negative score of IL18 in the mouse lung tissues.

Taken together, our results are consistent with the innate immune response mounted after viral infection. The scoring results that were not in agreement with the established roles of the factors can offer new insights into the regulatory mechanisms of inflammasome biology during viral infection. It would be important to score more viral infection datasets to be able to extract the regulatory traits of factors that were performed inconsistent with their conventional roles in inflammation, such as IRAK1, NF*κ*B, AHR, and P2RX7. Such regulatory traits will provide valuable information for elucidating the specific contribution of these factors during infection-provoked inflammation.

#### 3.2.2. Inflammasome Activation during Osteoarthritis and Rheumatoid Arthritis

**Figure 2:**
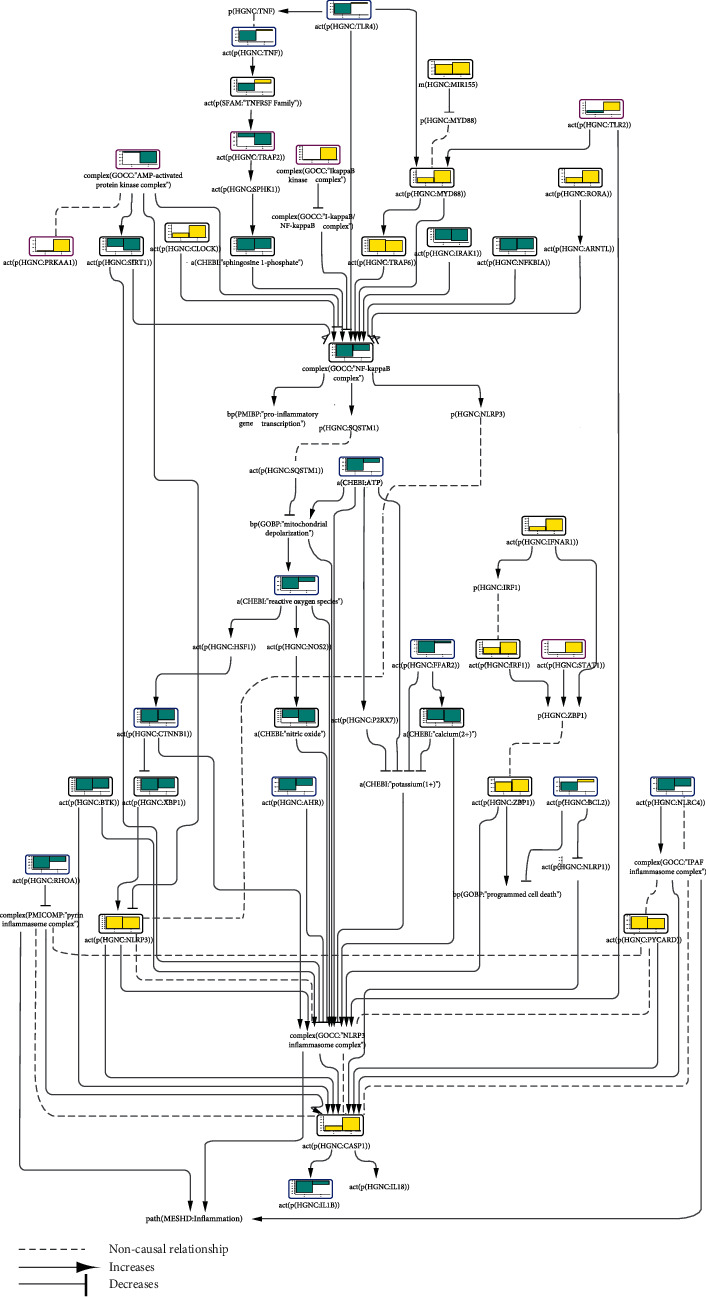
Part of the inflammasome network model scored with transcriptomic data from GSE55235. The bar graph above each node, which was scored, shows the inferred fold change in the synovial tissues of (1) osteoarthritis (OA) and (2) rheumatoid arthritis (RA) patients relative to healthy subjects. The directionalities are shown as yellow or blue bars for inferred upregulation or downregulation, respectively. The black outline refers to nodes that were significantly impacted in both diseases, the blue outline refers to nodes that were significantly impacted only in OA, and the purple outline refers to nodes that were significantly impacted only in RA.

Next, we chose GEO dataset GSE55235, where the transcriptomic data were derived from human synovial tissues from osteoarthritis (OA) and rheumatoid arthritis (RA) patients and healthy controls. RA is an autoimmune joint disease characterized by chronic synovitis that progresses to the destruction of cartilage and bone. The NLRP3 inflammasome contributes to RA pathogenesis and severity [48, 49]. OA is a joint disease where cartilage is damaged because of wearing away during aging [50]. Although commonly regarded as a wear-and-tear disease, there is evidence for an inflammatory component in OA [51, 52]. Indeed, our scoring results suggest that inflammasome components such as NLRP3, PYCARD (PYD and CARD domain containing), and CASP1 are activated in both diseases (Figure 2). NLRC4, an IPAF inflammasome component and stress sensor, was inferred to be significantly downregulated in the synovial tissues of OA patients. Interestingly, several inflammasome-activating factors—such as BTK, the NF*κ*B complex, and beta-catenin (CTNNB1)—as well as ROS, calcium cations, and ATP levels were inferred to be downregulated. Our network model has captured paths downstream of NF*κ*B and CTNNB1 that also lead to inflammasome inhibition. Even though it is an inflammasome priming factor, NF*κ*B restricts inflammasome activation through sequestosome 1 (SQSTM1, also known as p62), which promotes the clearance of damaged mitochondria [45]. While some studies suggest CTNNB1 to be an upstream promoting factor for the inflammasome [53, 54], CTNNB1 can also lead to inflammasome inactivation through inhibition of XBP1 (X-box binding protein 1), which, in turn, is an NLRP3 activator [55]. Interestingly, XBP1 was also inferred to be downregulated in both OA and RA samples. This downregulation of inflammasome-promoting factors suggests that other pathways are involved in inflammasome activation and such a counterbalance might be an effective measure for preventing excessive and uncontrolled inflammatory response. It is interesting to note that IL1B levels were also inferred to be downregulated in OA samples, and the IL18 score did not reach a significant value in any disease sample.

Consistent with the captured biology, all scored entities in the inflammasome-activating IFNAR1/IRF1/ZBP1 axis were inferred to be upregulated in both disease samples. Conversely, inhibitory factors of the NLRP3 inflammasome, such as the AMPK complex, SIRT1, and AHR, were inferred to be downregulated; the pyrin inflammasome inhibitor RHOA was also inferred to be downregulated.

While many upstream signaling molecules had similar inferred activities for both OA and RA, the TNFRSF (TNF receptor superfamily), TLR2, TLR4, TNF, STAT1, and BCL2 had opposite inferred scores, hinting at molecular mechanistic differences between the two diseases. All these entities were inferred to be upregulated in the synovial tissues of RA patients (the TNF, TLR4, and BCL2 scores did not reach statistically significant values for RA) and downregulated in OA patients' synovial tissues (the TLR2 and STAT1 scores did not reach statistically significant values for OA). Consistent with this finding, anti-TNF treatment has been reported to show beneficial outcomes in RA patients when compared with OA patients [56, 57].

#### 3.2.3. Inflammasome Network Model Scoring in Psoriasis

Psoriasis is a complex immune-mediated skin disease, which can also manifest as joint inflammation [58]. Psoriatic patients have a higher risk of comorbidities such as cardiovascular disease, inflammatory bowel disease, and nonalcoholic fatty liver disease [59–61]. There is a close link between this multifaceted disease and inflammasome activation; for example, an unbiased sequencing approach revealed inflammasome signaling as the highest differentially expressed pathway in psoriasis patients, whereby inflammasome activation was correlated with disease severity [62].

**Figure 3:**
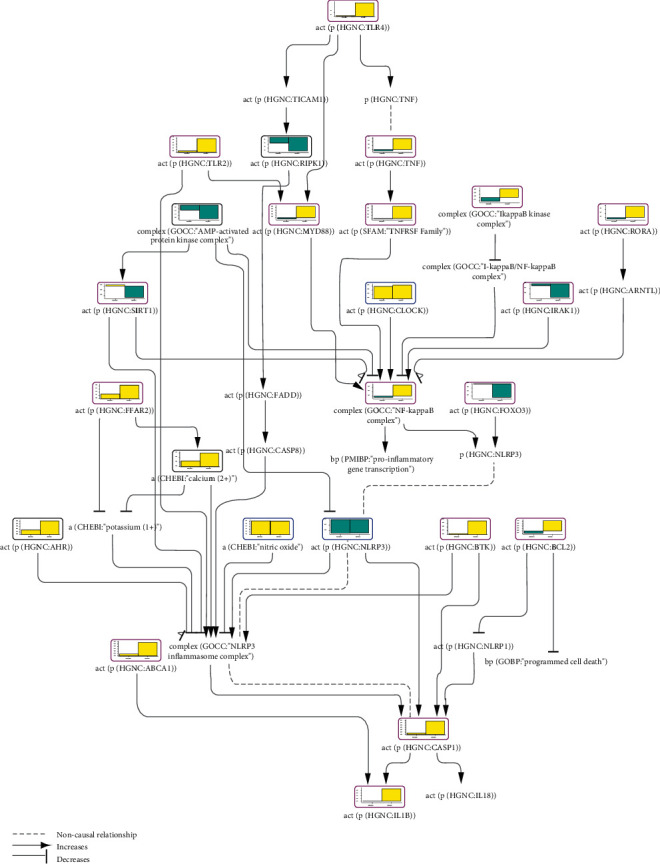
Part of the inflammasome network model scored with transcriptomic data from GSE2737. The bar graph above each node, which was scored, shows the inferred fold change in the (1) unaffected and (2) affected skin areas of psoriatic patients relative to the skin of healthy controls. The directionalities are shown as yellow or blue bars for inferred upregulation or downregulation, respectively. The black outline refers to nodes that were significantly impacted in both samples, the blue outline refers to nodes that were significantly impacted only in unaffected skin samples, and the purple outline refers to nodes that were significantly impacted only in affected skin samples.

We have scored the inflammasome CBN model with transcriptomic dataset GSE2737, derived from biopsy samples of affected and unaffected skin areas from psoriatic patients compared with skin samples from healthy control subjects. Evidently, the affected active disease skin areas had a higher number of significantly scored inflammasome-related nodes (Figure 3). Active CASP1 and IL1B were among the nodes enriched in the affected skin samples. NLRP3 activity, however, was not significantly inferred to be regulated in the active disease and was inferred to be downregulated in the unaffected skin areas. Nevertheless, a recent study in psoriatic patients found high expression levels of inflammasome sensors NLRP3, NLRP1, and AIM2 in peripheral blood cells as well as higher plasma levels of IL1B and IL18 [63]. A number of positive regulators of the inflammasome pathway—such as TLR2, TLR4, MYD88, the NF*κ*B complex, FFAR2, calcium cations, BTK, and the TNF/TNFRSF—were inferred to be significantly upregulated in psoriatic skin samples relative to healthy skin samples. TNF, also inferred to be upregulated, is a recognized hallmark of psoriasis [64], and anti-TNF therapy is an approved treatment for moderate-to-severe plaque psoriasis, where it helps normalize CASP1 activity and plasma IL1B and IL18 levels and reduce skin and joint lesions [63, 65, 66].

AMPK and SIRT1, both being factors that limit inflammasome activation, were inferred to be downregulated in psoriatic patient skin samples, concordant with downstream inflammasome activation. Next, the antiapoptotic protein BCL2 was inferred to be upregulated in affected skin biopsy samples. A study from Iizuka et al. suggests premature keratinocyte death in psoriasis [67], and our scoring result in this case could be a part of a compensatory mechanism. Inconsistent with their inflammasome-activating roles, IRAK1, RIPK1 (receptor-interacting serine/threonine kinase 1), and FOXO3 (forkhead box O3) were inferred to be downregulated. This could be explained as a countervailing attempt of the affected tissue to halt excessive inflammation. The inferred upregulation of the inflammasome-inhibiting AHR can be explained in a similar fashion.

Overall, the inflammasome network model scoring with transcriptomic data from the skin samples of psoriatic patients was largely consistent with the curated literature findings. This holistic view of the network of pathways helps identify the routes that are highly involved in inflammasome activation and distinguish the ones that are not involved or are antagonizing to inflammatory response, thus informing potential drug treatment efforts.

#### 3.2.4. Inflammasome Network Model Scoring in Aging Mouse Liver

In recent years, the inflammasome has emerged as an important factor implicated in metabolic and age-related diseases [68]. AMPK, a central molecule in energy metabolism that is also involved in processes of aging, and SIRT1, a key factor implicated in aging, are among the upstream modulators of NLRP3 inflammasome activation. Of note, the activities of both these proteins were inferred to be downregulated in our network model scoring with transcriptomic data from SARS MA15 virus-infected mouse lungs and synovial tissues of arthritis patients. A very recent study on chronic viral infections suggests a common signature of immune dysfunction in viral infections and aging-related inflammation [69].

**Figure 4:**
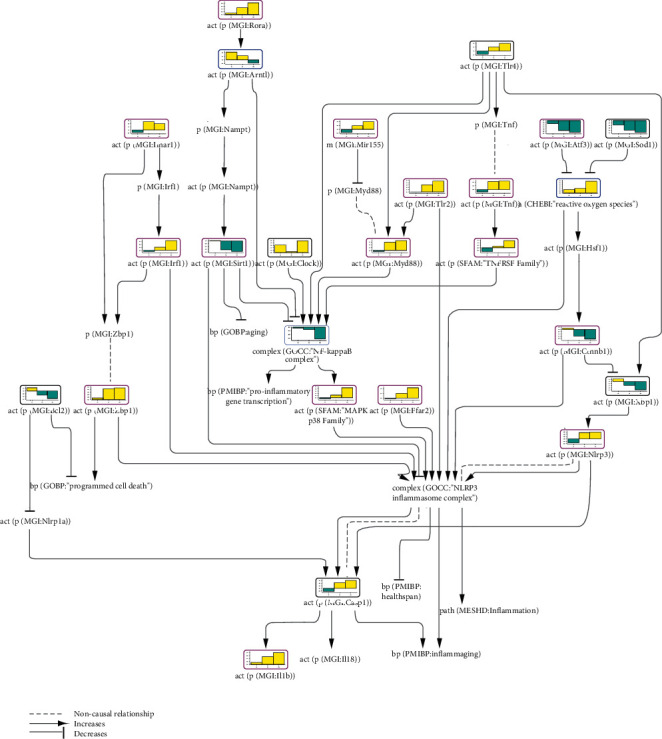
Part of the inflammasome network model scored with transcriptomic data from E-MEXP-839. The bar graph above each node, which was scored, shows the inferred fold change in the liver tissue of (1) 16-, (2) 96-, and (3) 130-week-old wild-type (WT) mice relative to 8-week-old WT mice. The directionalities are shown as yellow or blue bars for inferred upregulation or downregulation, respectively. The black outline refers to nodes that were significantly impacted in all ages, the purple outline refers to nodes that were significantly impacted at two time points, and the blue outline refers to nodes that were significantly impacted at only one time point.

With an aim to determine inflammasome CBN model perturbations during a lifespan, we analyzed the E-MEXP-839 dataset from the ArrayExpress database. This dataset compares the mouse liver transcriptomic profiles of adult 16-, 96- and 130-week-old wild-type (WT) C57Bl/6J mice with those of 8-week-old WT mice. Mice at the age of 16 weeks are generally not affected by senescence, whereas mice over 24 months of age are considered very old and show apparent histological lesions associated with aging [70]. Accordingly, we identified only six iNodes that were significantly impacted at all three ages; moreover, all iNodes that were inferred to be significantly regulated at two time points out of the three showed significant scores for the latter, that is, 96- and 130-week time points, with the exception of the TNFRSF family iNode, which was inferred to be down- or upregulated in the liver of 16- and 130-week-old WT mice, respectively. In Figure 4, we show a subnetwork of the inflammasome activation CBN model, including iNodes, that reached a statistically significant score in the mouse liver in at least two life stages. Some nodes that were inferred to be regulated at only one time point—such as ARNTL, the NF*κ*B complex, and ROS—were included because of their bridging position in pathways leading to inflammasome activity modulation. Analysis of the last two time points showed clear inferred upregulation of proinflammatory entities such as IFNAR1, IRF1, ZBP1, TLR2, TLR4, MYD88, TNF, and the MAPK p38 family, inflammasome constituents NLRP3 and CASP1, and the inflammasome product, active IL1B. However, some proinflammatory factors were inferred to be downregulated, such as the NF*κ*B complex (significant score only in 130-week-old mice) and XBP1. Conversely, we observed inferred downregulation of anti-inflammatory, antiaging molecules, such as SIRT1 and SOD1 (superoxide dismutase 1). The inferred scores of AMPK did not reach statistical significance at any time point. Concomitant with the decrease in SOD1 activity, ROS levels increased with age, creating an aging-accelerating milieu due to accumulated damage [71]. Furthermore, from our CBN scoring data, we could conclude that liver cell death rate increased with age, as the activity of the antiapoptotic protein BCL2 was inferred to be downregulated at later life stages of the mice. Additionally, the activity of ZBP1, which is involved in PANoptosis, was inferred to be increased in 96- and 130-week-old WT mice. PANoptosis refers to collective activation of pyroptosis, apoptosis, and necroptosis, which can result, for example, from ZBP1 pathogen sensing [72–74].

This perturbation of multiple inflammatory pathways in old animals supports the crucial role of inflammation in aging processes. Our network model scoring methodology can potentially offer new targets for intervention for healthy and slow aging approaches.

#### 3.2.5. Inflammasome Response to Potassium Treatment

**Figure 5:**
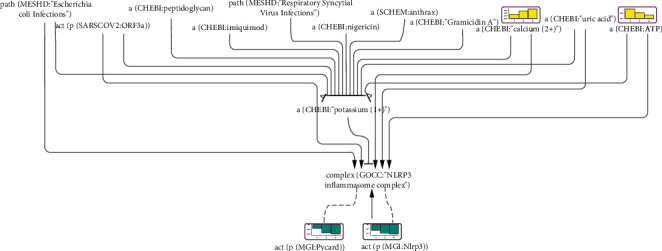
Part of the inflammasome network model scored with transcriptomic data from GSE11258. The bar graph above each node, which was scored, shows the inferred fold change in mouse hippocampus after (1) 1 hour, (2) 3 hours, and (3) 6 hours of 50 mM KCl treatment. The directionalities are shown as yellow or blue bars for inferred upregulation or downregulation, respectively. The black outline refers to nodes that were significantly impacted at all time points, and the purple outline refers to nodes that were significantly impacted at two time points.

In all previous examples here, inflammasome components were inferred to be activated, as expected. We next asked if the network model would react to a stimulus that is supposed to block inflammasome activity. Many stimuli converge on potassium efflux, which is an essential mechanism of inflammasome activation [75, 76]. We chose a dataset of potassium treatment in murine hippocampal neurons (GSE11258), where inflammasome activity was expected to be blocked. Many studies confirm the presence and activity of the NLRP3 inflammasome in the hippocampus [77–79]. Indeed, treatment with 50 mM KCl for 1, 3, or 6 h causes a time-dependent decrease in the inferred activities of inflammasome components such as NLRP3 and PYCARD (significant scores at 3 and 6 h of potassium treatment) (Figure 5). The CASP1 score did not reach statistical significance. A snapshot of the inflammasome network model shown in Figure 5 includes several infectious agents and chemicals that trigger potassium efflux and consequently activate the inflammasome. Some of them also contribute to inflammasome activation independent of potassium concentration, for example, through NF*κ*B-induced NLRP3 gene transcription during the inflammasome priming step. Presumably, higher intracellular potassium levels can block the inflammasome-triggering potential of these stressors. An example of this blockage is shown in a study by Suzuki and colleagues, where an increase in extracellular potassium concentration inhibited inflammasome-dependent IL1B production triggered by lipopolysaccharide and ATP treatment [80]. Both ATP (significant scores at 1 and 3 h of potassium treatment) and Ca^2+^ (significant scores at 3 and 6 h of potassium treatment)—which are upstream negative regulators of potassium levels in our network model—were inferred to be upregulated, likely to counteract the burden of KCl salt exposure. Through the purinergic receptor ATP-gated channel P2RX7, ATP can act as a potassium efflux agent, enabling the influx of calcium and sodium cations to change the membrane potential, which, in turn, facilitates potassium efflux through KCNK6 (potassium two pore domain channel subfamily K member 6, also known as TWIK2) [80, 81].

## 4. Conclusion

We have presented here a CBN model that describes inflammasome activation on the basis of causal molecular relationships extracted from pertinent research articles. We also scored the inflammasome model with transcriptomic data derived from a viral infection of the lungs, osteo- and rheumatoid arthritis, psoriasis, and aging. In all these diverse conditions, the pathways leading to NLRP3 inflammasome activation were significantly impacted, implying inflammasome involvement. More interestingly, some upstream modulating paths were differently affected in individual datasets, providing insights on disease- or condition-specific inflammasome regulation. It is important to emphasize that the results presented in this report are from a small number of studies, and the network scoring needs to be repeated with several other datasets to support our findings. Regardless, the approach is very powerful for generating new hypotheses that, once experimentally verified, could lead to the discovery of new and more efficient therapeutics against conditions that engage inflammasomes, such as viral infections and inflammatory and metabolic diseases. We are continuing these efforts by expanding the suite with network models for other relevant components of innate and adaptive immune biology, including neutrophils, macrophages, dendritic cells, natural killer cells, and B cells.

Currently, of the 120 potentially inferable nodes (protein and protein family activities, complexes, and microRNAs as well as some chemicals) in the inflammasome CBN model, only 55 are iNodes; that is, only 55 nodes have the downstream transcript layer populated with data from public datasets. As a consequence, more than half of the inflammasome network model backbone nodes were left out of the model scoring and discussion in this study. Future work will complement the current results and might lead to new exciting conclusions, especially toward explaining the causal inconsistencies in the affected pathway scores. The results shown here are just a glimpse of what model scoring can offer, and the true value will be in the analysis of noninvasive human samples (e.g., blood) for evaluating the systemic component in different disease stages and toxic exposures and upon drug treatment or postvaccination responses.

## Figures and Tables

**Table 1:** BEL vocabulary for the namespaces used in the inflammasome CBN model.

*BEL function*	*BEL function expanded*	*Example*

a	Abundance	a(CHEBI:“calcium(2+)”)
act	Activity	act(p(HGNC:NLRP3))
bp	Biological process	bp(GOBP:autophagy)
complex	Complex	complex(GOCC:“NLRP3 inflammasome complex”)
m	MicroRNA	m(HGNC:MIR146A)
p	Protein	p(HGNC:NLRP3)
path	Pathology	path(MESHD:Sepsis)
r	RNA	r(HGNC:IL1B)
Sec	Secretion	sec(p(HGNC:IL18))

*Namespace identifier*	*Namespace identifier expanded*	*Example*
HGNC	HUGO gene nomenclature committee	p(HGNC:TLR4)
MGI	Mouse genome informatics	p(MGI:Tlr4)
RGD	Rat genome database	p(RGD:Tlr4)
SARSCOV2	Severe acute respiratory syndrome coronavirus 2	p(SARSCOV2:E)
CHEBI	Chemical entities of biological interest	a(CHEBI:“nitric oxide”)
SCHEM	Selventa legacy chemical names	a(SCHEM:“Toxin B, Clostridium difficile”)
GOBP	Gene ontology biological process	bp(GOBP:“mitochondrial depolarization”)
GOCC	Gene ontology cellular components	complex(GOCC:“NF-kappaB complex”)
SCOMP	Selventa named complexes	complex(SCOMP:“CHRN Complex”)
SFAM	Selventa named protein family	act(p(SFAM:“TNFRSF Family”))
MESHD	Medical subject headings	path(MESHD:Inflammation)
PMIBP	Custom namespace for biological processes	bp(PMIBP:Inflammaging)
PMICOMP	Custom namespace for complexes	complex(PMICOMP:“pyrin inflammasome complex”)
PMIDIS	Custom namespace for diseases	path(PMIDIS:“influenza A virus infection”)
PMIPFAM	Custom namespace for protein families	p(PMIPFAM:“TLR Family”)

## Data Availability

The inflammasome network model is available in the CBN database (http://causalbionet.com/).
